# For whom the bell tolls: periodic reactivation of sensory cortex in the gamma band as a substrate of visual working memory maintenance

**DOI:** 10.3389/fnhum.2014.00696

**Published:** 2014-09-04

**Authors:** Marieke Karlijn Van Vugt, Ramakrishna Chakravarthi, Jean-Philippe Lachaux

**Affiliations:** ^1^Artificial Intelligence, Cognitive Modeling Group, University of GroningenGroningen, Netherlands; ^2^School of Psychology, University of AberdeenAberdeen, UK; ^3^U1028, INSERMLyon, France

**Keywords:** gamma oscillations, working memory, working memory capacity, attention, ECoG

## Abstract

Working memory (WM) is central to human cognition as it allows information to be kept online over brief periods of time and facilitates its usage in cognitive operations (Luck and Vogel, 2013). How this information maintenance actually is implemented is still a matter of debate. Several independent theories of WM, derived, respectively, from behavioral studies and neural considerations, advance the idea that items in WM decay over time and must be periodically reactivated. In this proposal, we show how recent data from intracranial EEG and attention research naturally leads to a simple model of such reactivation in the case of sensory memories. Specifically, in our model the amplitude of high-frequency activity (>50 Hz, in the gamma-band) underlies the representation of items in high-level visual areas. This activity decreases to noise-levels within 500 ms, unless it is reactivated. We propose that top-down attention, which targets multiple sensory items in a cyclical or rhythmic fashion at around 6–10 Hz, reactivates these decaying gamma-band representations. Therefore, working memory capacity is essentially the number of representations that can simultaneously be kept active by a rhythmically sampling attentional spotlight given the known decay rate. Since attention samples at 6–10 Hz, the predicted WM capacity is 3–5 items, in agreement with empirical findings.

## Introduction

Working memory (WM) allows us to keep information online over brief periods of time and use it in cognitive operations (Luck and Vogel, 2013). To this day, it has proven difficult to reconcile theories of WM inspired by behavioral data (psychological theories) and those by brain measurements (neural theories), despite clear areas of convergence. We will briefly outline some of the main ideas and difficulties on both sides in the context of WM for sensory information and propose a possible interface between these approaches.

### Psychological theories of WM

Despite several differences, most psychological theories of WM agree on the necessity for an active mechanism to prevent spontaneous decay of the representations of sensory items: it is thought that items that are not transferred to a more durable long-term store decay over time (e.g., Raaijmakers and Shiffrin, 1981). Preventing memory decay could occur by placing the items into a special store (e.g., Baddeley, 2003), or by holding them in the focus of attention and periodically reactivating them (e.g., Anderson, 1983; Cantor and Engle, 1993; Cowan, 1995).

The idea that attention is critical for reactivating memory traces has become a central point of discussion. Many reports (e.g., Awh et al., 1998; Downing, 2000; Awh and Jonides, 2001; Jha, 2002; Theeuwes et al., 2011 among others) indicate for instance that attention is preferentially deployed to remembered locations even during the maintenance period, when stimuli are no longer physically present. Similarly, disrupting attention during the maintenance period negatively impacts WM for remembered locations (Awh et al., 1998). However, others disagree with the idea that WM and attention share a common resource (e.g., Woodman et al., 2001; Johnson et al., 2008). Woodman et al. (2001) found that loading WM to its capacity did not affect visual search efficiency, a measure of attention, contradicting a prediction by the common resource hypothesis. However, there might be some methodological issues with these studies. For example, the Woodman et al. (2001) study used clustered stimuli to test their contention that WM and visual attention do not interact. In contrast, Anderson et al. (2013) in a similar dual WM-visual search paradigm, showed that no interference between WM and visual search occurs only if objects in the search task are grouped or clustered stimuli, perhaps because attention can search more efficiently in such situations. In fact, Johnson et al. (2008) themselves found that increasing WM load increases reaction times when participants search for an object that is a combination of two features (i.e., when binding is required) but not when such binding is not required. This suggests that WM load interferes with a task that requires attention such as binding (Treisman and Gelade, 1980). Further, Anderson et al. (2013) tested various kinds of stimuli and showed that visual search efficiency is closely related to WM capacity, indicating that they share a common resource. Other studies have also shown mutual interference between visual search and WM tasks (Oh and Kim, 2003, 2004; Woodman and Luck, 2004).

Based on such findings and their own, Barrouillet et al. (2004), have taken the idea of attentional involvement in WM maintenance one step further. They posit a periodic, rather than continuous, process of reactivation by attention. This is based on four tenets and observations they make: (1) WM processing and maintenance requires the use of a limited attentional resource; (2) when attention switches away, items are subject to decay; (3) attention-demanding cognitive processes therefore prevent refreshing of memory traces; and (4) there is only one attentional process at a time, and thus there should be rapid switching between processing and maintenance aspects of a task (Barrouillet et al., 2007). Taken together, it can be argued that items in WM decay with time, and a resource-limited process, attention, rapidly shifts between these decaying representations in order to reactivate them. In this paper, we expand on this explanation, grounding it in neurophysiological evidence that places constraints on such a theory.

### Neuroscience of WM

There is also considerable debate about what neural system underlies WM. Baddeley has associated WM with right fronto-parietal areas for visual memories and with left fronto-parietal areas for verbal memories (Baddeley, 2003). The idea that the frontal lobe, and in particular the prefrontal cortex (PFC), is involved in maintenance has been substantiated by recordings made by Goldman-Rakic and others (Goldman-Rakic, 1995), who observed that some PFC neurons have increased and sustained activity over the course of an interval in which a stimulus had to be maintained. Even recently, Salazar et al. (2012) found clear evidence for item-specific increases in fronto-parietal 14–30 Hz beta oscillatory coherence during WM maintenance. Several other studies have shown that lower-frequency oscillatory synchronization between prefrontal and temporal lobes (Von Stein et al., 1999; Serrien et al., 2004) and between prefrontal and parietal lobes (Lutzenberger et al., 2002; Babiloni et al., 2004; Kopp et al., 2006; Payne and Kounios, 2009) are associated with the maintenance of information during WM tasks. Some of these effects are also dependent on the number of items that participants have to maintain (see Fell and Axmacher, 2011, for a discussion). In addition to involvement of individual oscillatory bands in WM maintenance, phase-amplitude coupling in PFC between low and high frequency oscillatory components has also been observed in the context of WM tasks (Siegel et al., 2009), and in humans one study has further shown that the magnitude of this phase-amplitude coupling is associated with a person’s WM capacity (Sauseng et al., 2009).

Although there is a large body of evidence implicating the PFC in WM maintenance, there is also ample evidence that other regions, including the hippocampus (Axmacher et al., 2007; van Vugt et al., 2010), are involved, especially for visually complex items. However, studies emphasizing the hippocampus typically do not consider the role of attention in WM, which as discussed above, is crucial. If we assume the PFC is involved in (sensory) WM, one central issue is whether PFC neurons actually contain a memory trace of the maintained items, as early studies showing continuous, item-specific activations might have suggested (Goldman-Rakic, 1995). This view is problematic for several reasons: (a) it implies a strong redundancy between PFC and sensory areas, since every item which can be perceived and identified in sensory cortices should once again be represented by a neural group in the PFC, in a one-to-one fashion, during memory maintenance. This duplication seems “metabolically costly” (Sreenivasan et al., 2014); (b) PFC neurons do not exhibit the same degree of selectivity as sensory neurons, which suggests that potential confusions between different, but closely resembling, sensory items could occur in the PFC if memory traces were stored there; that is, if two items which can be differentiated in sensory cortices were maintained by the same pattern of neural activation in the PFC, how could they be differentiated in the recall phase? (e.g., Miyashita and Chang, 1988); and (c) the evidence that PFC supports the representation of items in WM are derived from animal experiments with extensive training and exposure to the same sensory stimuli, which is very unlike the situations in which humans usually use WM. Consequently, it cannot be excluded that specific PFC neural ensembles might specialize over the training phase to store each element of the limited set of items used in the experiment. Obviously, such one-to-one mapping could not occur to store novel and unique sensory items seen only once in real-life situations.

Yet, it is clear that *both* PFC and sensory areas participate in maintenance of items in sensory WM, but what are their respective contributions? To avoid any loss of specificity and selectivity during the maintenance period, the actual representation of sensory items must necessarily be stored where it was generated in the first place during stimulus presentation: in sensory areas themselves. If so, it immediately raises two (possibly related) questions: (a) how are the representations maintained over time in sensory areas; and (b) what is the role of the PFC? In the following, we propose a model that directly addresses these two questions.

### The “hammer and bells” model

Any model of sensory WM must include a mechanism of reactivation to avoid neural representations of memory items to decay to noise level. Such mechanism can either be local (self-maintenance in sensory areas), or global, via long-range projections from distant cortical areas. However, both possibilities have their problems.

### Problems with local reactivation

Some authors have proposed that reactivation occurs locally, for instance with the aid of recurrent interactions in the local neural architecture (Lisman and Idiart, 1995; Luck and Vogel, 2013). However, local reactivation by itself does not explain why some items are stored in WM while others are not, usually depending on current behavioral goals and/or task instructions (e.g., Sperling, 1960). Rather, we propose that the special status of remembered items is largely decided by remote cortical areas supporting memory of current behavioral goals (i.e., task sets), namely in the PFC (Sakai and Passingham, 2003), in agreement with Sreenivasan et al. (2014).

### Problems with a long-range reactivation

**Figure 1:**
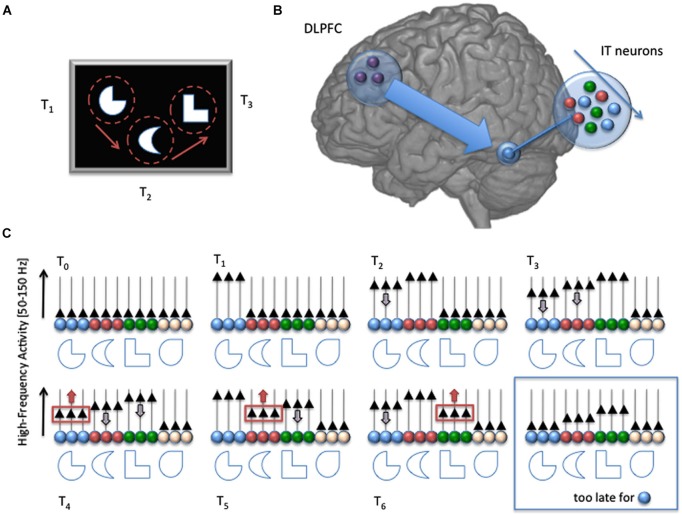
**Schematic description of our proposed mechanism for sensory working memory maintenance**. At time T_0_, a set of to-be-memorized stimuli appears on screen. The stimuli are attended sequentially, through successive overt attention shifts in this example (at time T_1_, T_2_ and T_3_). Each fixation triggers a strong response of the neural assembly (in the Infero Temporal Cortex in this example, color coded), which acts as a specific detector for the stimulus receiving attention (increase of High-Frequency Activity [HFA; 50–150 Hz], black triangles). But as attention goes away, the activity of the neural assembly starts decreasing with a slope characteristic of sensory cortical areas (gray arrow pointing down). Such decrease is compensated by the long-range action of DLPFC neurons, which select regularly the weakest active assembly and get it back to its original value (red arrows pointing upward at T_4_, T_5_ and T_6_), providing a rhythmic, circular maintenance mechanism. If the DLPFC fails to reactivate a neural assembly, its activity falls down to baseline level and cannot be distinguished anymore from the other, inactive, assemblies (i.e., white neurons, blue box, bottom right). With such mechanism, DLPFC neurons don’t need to store a precise and unambiguous identifier of the neural assemblies they must keep active, avoiding the need for a duplicate of all possible sensory representations in the prefrontal cortex. All that is needed is a mechanism to select the weakest active assembly and promote its activity, in combination with object-based attention. The memory span depends on the time it takes for a HFA in a neural assembly to decay spontaneously to baseline level, and on the time it takes for DLPFC neurons to shift their focus from one assembly to another (similar to a shift of attention). Note that although the neural assemblies are considered separate in this simplistic schema, the model can easily be extended to overlapping neural representations. Also, the top-down influence from the DLPFC is not necessarily direct, and could involve the parietal lobe.

If reactivation of neural assemblies in sensory cortex is directed by distant cortical regions, say the PFC, then PFC neurons that are responsible for the reactivation of the to-be-maintained sensory items must unambiguously identify and connect to the exact set of sensory neurons that support those items, with perfect selectivity. This constraint raises, again, the problem of a possible redundancy between PFC and sensory cortices, wherein each possible sensory item is tagged by a specific neural group in the PFC (see above). Then, the PFC could, by itself, be the substrate of the memory trace, and thus duplicate sensory areas with all associated contradictions (Postle, 2006; Lee et al., 2013). One route of escape from that dead-end, however, is that the neural representations of remembered items in sensory areas have a distinct feature: they are presumably more active than other nearby, sensory neurons, at least before full decay, Therefore, a full redundancy between PFC and sensory areas is only needed to reactivate a memory trace if it has decayed to noise level; until then, PFC neurons can selectively dissociate neurons which participate in the memory trace based on their higher activity. In short, residual activity in the sensory areas could be read by PFC neurons as the signature of to-be-reactivated neurons. A straightforward mechanism, therefore, for WM maintenance, would be to simply reactivate all sensory neurons that are *currently* active (Figure 1).

### A bell analogy

**Figure 2:**
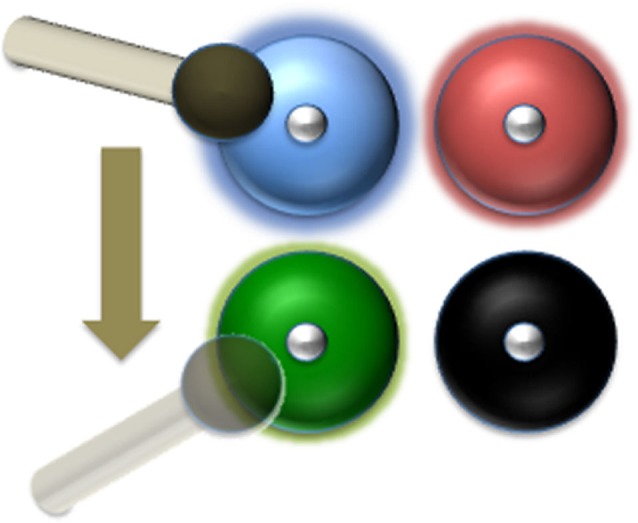
**The hammer-and-bells analogy: a blind drummer just hit the blue bell**. Next, he goes for the bell with the faintest ring (the green one in this case). He can’t find the black one any more because it has gone silent. The number of bells that the drummer can keep ringing depends on the time it takes for the sound of each bell to fade away and the time it takes the drummer to shift the hammer from one bell to another. In our model, each bell corresponds to a neural assembly storing the representation of one item in WM. The “sound” of each bell is the High-Frequency Activity produced by that neural population, which must be reactivated before going back to baseline level (silence). The blind drummer might correspond to the DLPFC storing the instruction to keep the items in WM.

Given the need for periodic reactivation (from both the neuroscientific and psychological theory perspectives), we propose a different mechanism directly inspired by a practical, physical problem (Figure 2). Consider a set of bells. If one decided to hit a subset of them with a hammer, and keep doing so, so that the sound of each bell of that subset never dies, how should one proceed? An obvious solution would be to remember which bells must be hit, and hit them in a sequence, repeatedly. But that would amount to having a separate, independent memory trace of which bells must be hit. If we want to think of the bells as the primary memory traces in the sensory areas, this possibility amounts to the one we discussed above and would raise the obvious objection: why have two memory traces (one, a set of vibrating bells, and the other the memory in the bell-player), why not just the second, “prefrontal” one? To solve that problem without an additional memory trace, we need to find a way to identify which bells must be hit on the sole basis of what distinguishes them from other bells, namely the fact that they are still ringing. The only solution, then, is to hit all the bells that are still ringing, as they would become undistinguishable from the rest once silent. There is no need to remember which specific bell was active. All that is needed is a system that monitors if a bell is still vibrating, and if so, hits it. This means that the number of bells that can be kept ringing simultaneously depends on two parameters: the time it takes to shift the hammer from one bell to another, and the time it takes for the sound to decay, in each bell. We can then predict the maximum number of bells that one can actively maintain. In our model, the bells would be in the sensory areas and PFC neurons would hold the hammer. Our analogy predicts that we can predict the number of sensory items that can be maintained in WM from the time it takes for their neural representation to decay to noise level and from the time necessary for PFC neurons to reactivate one trace after another.

### Role for attention

If there is indeed such periodic reactivation, what neural mechanism might reactivate these fading representations? The candidate mechanism should have at least two properties: (a) it should be selective, to enhance active neural representations in sensory cortex while not activating representations of concurrent items; and (b) it should take into account current behavioral goals. These two properties almost define attention, as it is currently understood. In line with that remark, attention is known to be intimately involved in the maintenance of objects in WM (Awh and Jonides, 2001; Jha, 2002; D’Esposito, 2007). Several researchers (Cowan, 1988, 1995; Barrouillet et al., 2004; RepovŠ and Baddeley, 2006; but see Fougnie, 2008) have even posited a cognitive model where spatial WM is merely the information currently present within the focus of attention. When selected, attention enhances activation of that representation (Fries et al., 2002). Attention is also intimately involved in modulating activity based on behavioral goals (e.g., Connolly et al., 2002; Corbetta et al., 2008; Leber, 2010; Chun et al., 2011). Moreover, as desired for periodic reactivation, recent evidence suggests that attention acts in a rhythmic manner (VanRullen et al., 2007; Busch and VanRullen, 2010; Landau and Fries, 2012), sampling the input stream at around 6–10 Hz. Taking all these considerations together, attention could serve as a mechanism by which fading representations can be reactivated.

### Maintaining several items simultaneously

This reactivation model based on the bell analogy also solves another problem, that is, the mechanism by which several items can be simultaneously maintained. If there is WM in sensory areas, then apart from the question of reactivating these decaying WM representations, another important question is: how do neural processes maintain multiple items *simultaneously* in WM? Suppose that in sensory areas each item *i* has activated, when presented, a neural population P_*i*_. Then the question is how can the neural activity of all populations P_*i*_ be maintained during the delay period? One easy solution would be for attention to sustain that activity simultaneously in all P_*i*_, which implies that a PFC-mediated attentional modulation could target all P_*i*_ simultaneously. However, there is a long-standing debate about whether attention can select multiple objects simultaneously or whether attention has a unitary focus and switches between objects (LaBerge and Brown, 1989; Posner and Petersen, 1990; Castiello and Umiltà, 1992; Bichot et al., 1999; Müller et al., 2003; Howe et al., 2010). Recent evidence suggests that even when attention appears to be selecting several items simultaneously–the so called “divided attention” model–its is in fact selecting one object at a time and rapidly switching between different objects (VanRullen et al., 2007; Hogendoorn et al., 2010). Based on these findings, we suggest that decaying items are sequentially reactivated by a unitary attentional spotlight.

Given the evidence that items are selected sequentially, what consequences does that have for maintenance in WM? One fallout is that attention would also be processing and hence reactivating items in WM sequentially. Another is that, since attention samples sequentially, all items are loaded into WM sequentially, even if multiple to-be-remembered items are presented simultaneously. This might, to some extent, prevent the representations of multiple active items being “confused” with each other. A neural population that is most active at a given time represents one item. The items are separable because they are all not simultaneously active to the same extent. If multiple representations were simultaneously equally active, then the brain would have no way to distinguish whether those were a single object or two separate ones, since both are likely to be represented in overlapping neural regions. This predicts that high-contrast objects that take longer to decay could potentially be confused with low-contrast objects represented by the same neural population that are presented at a later time (Singer and Gray, 1995).

Various methods to measure the duration of attentional shifts between objects have come up with a number of about 100–300 ms (4–10 Hz). Chakravarthi and VanRullen (2011b) showed that this “shifting” of attention occurs even when a single object is attended–attention samples the object at around 7 Hz (also see VanRullen et al., 2005). That is, attention selects an object and disengages from it periodically, even when sustained attention is required for the task. Recent work has further supported this hypothesis (Landau and Fries, 2012; Song et al., 2014). In summary, this single mechanism of periodically sampling attention could reactivate both single and multiple items to be maintained in sensory WM.

Periodic attention directed by PFC in combination with reactivating decaying memories in sensory areas could therefore explain WM maintenance and impose a limit on the number of items that can be maintained in WM. If we assume that memories are represented by gamma-band synchronized assemblies in sensory areas (Luck and Vogel, 2013) then we should be able to infer from gamma-band recordings the maximal waiting time between two reactivations of the same neural populations. In addition to this, if we have an estimate of the time required by attention to reactivate separate neural populations (that is, the attentional “switch time”), we can compute the maximum number of items that can be stored in WM. Here we will present iEEG (intracranial electroencephalography) data that address this issue.

### Empirical data

**Figure 3:**
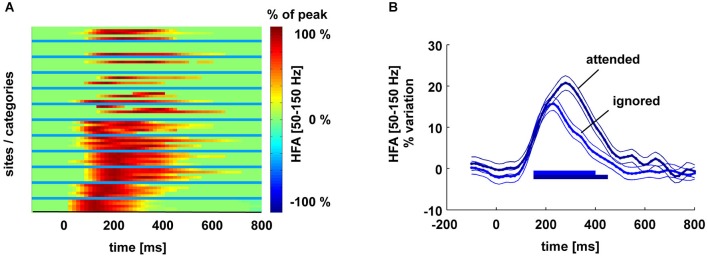
**Panel (A) shows the temporal profile of HFA responses to visual stimuli in six participants in high and low-level visual areas during a simple visual oddball task in which participants watched stimuli from different categories (see Vidal et al. (2010) for a detailed description of the protocol)**. Response duration was inferred from the duration between the peak response and the last 50-ms window in which amplitude departed significantly (Bonferroni corrected, *p* < 0.05) from baseline. The matrix (left) shows for each site and for each stimulus category (between blue lines), the HFA response as a function of time. Amplitude is expressed as % of the response peak. All time points that do not significantly differ from the pre-stimulus baseline have been set to 0. Sites range from early (bottom) to high-level (top) visual areas. Each row within a given area represents activity in response to a different stimulus. Panel **(B)**: HFA response in the fusiform gyrus to attended vs. ignored words during an attentive reading task. The main effect of attention is to prolong response duration. Horizontal lines indicate HFA values significantly above baseline level (−100–0 ms).

Intracranial recordings reveal a spontaneous decay of gamma-band neural activity after a presented stimulus is removed. Figure 3A shows the temporal profile of High Frequency Activity (HFA) responses to visual stimuli in six participants in several visual areas during a simple visual oddball task in which participants watched stimuli from different categories (see Vidal et al. (2010) for a detailed description of the protocol). The total duration of HFA, the “response duration”, was calculated as the duration between the peak onset and the last 50-ms window in which amplitude departed significantly (Bonferroni-corrected, *p* < 0.05) from baseline. The matrix (left) shows the HFA response as a function of time for each site (between blue lines) and for each stimulus category (within blue lines). Each row within a given area represents activity in response to a different stimulus. Amplitude is expressed as a percentage of the response peak. All time points that do not significantly differ from the pre-stimulus baseline have been set to 0. Sites range from early (bottom) to high-level (top) visual areas. This shows that the timing of decay is remarkably consistent across these areas; response duration is, on average, 450 ms (SD 80 ms; range 300–600 ms).

We also observe in these iEEG data a mechanism that prevents decay: attention. Figure 3B shows the HFA response in the fusiform gyrus to attended vs. ignored words during an attentive reading task. The main effect of attention is to prolong response duration even if the visual stimulus triggering that activity is no longer present. Although a local origin of this attentional effect is possible in principle (local recurrent interactions acting to sustain neural activity), it is likely to be triggered directly or indirectly by executive brain areas, presumably in the prefrontal cortex, where task instructions are known to be represented (task-set neural ensembles in the lateral prefrontal cortex, PFC). This is consistent with the findings that top-down influences, such as mental imagery (Hamamé et al., 2012) and attention (Jung et al., 2008), have been shown to sustain or even generate HFA de novo. These findings suggest that attention could reactivate HFA, and thus refresh items in WM.

**Figure 4:**
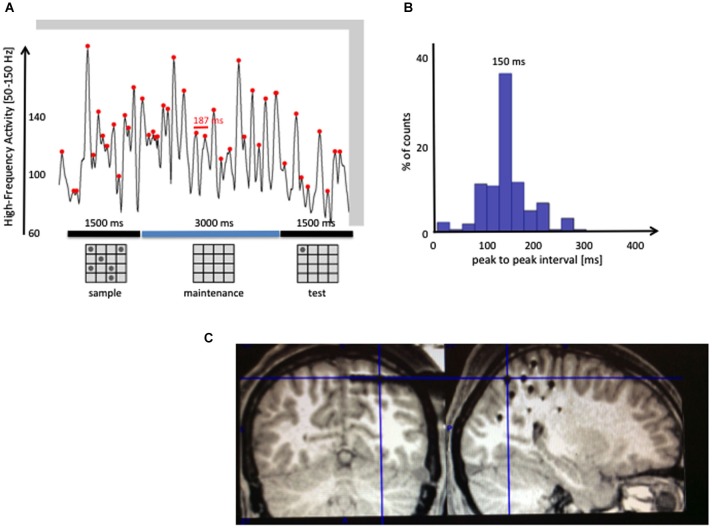
**Rhythmic modulations of gamma band activity during the maintenance of sensory items**. High-Frequency Activity [HFA; 50–150 Hz] was recorded in the dorsal visual stream of an epileptic patient while she was performing a visuo-spatial WM task (the MRI slice in panel **(C)** shows with crosshairs the location of the depth-electrode of interest). The patient was instructed to remember a set of six locations on a 4 × 4 grid, shown for 1.5 s. After a 3 s maintenance interval, one dot (test) appeared and the patient had to decide whether it was among the six locations shown in the sample stimulus. Panel **(A)** shows one illustrative trial, with red dots on each local maximum of the high-frequency envelope (defined as the maximum value in a sliding 80 ms window). Intervals between consecutive peaks (i.e., 187 ms, in red) were then measured in all trials of the experiment (*N* = 12) to generate the histogram shown in **(B)**. The distribution of intervals shows a clear accumulation between 100 and 200 ms, centered around a peak at 150 m. This distribution is consistent with a mechanism generating periodic peaks of HFA between 6 and 10 Hz, even in the absence of novel sensory inputs.

While Figure 3 shows only one bell strike, Figure 4 shows an example of possible successive bell strikes in the absence of a novel sensory input. HFA was recorded along the dorsal visual stream of an epileptic patient (using iEEG), while she had to maintain in memory a set of six spatial positions (6 dots on a 4 × 4 grid; see panel a). On top of a slower modulation, the trace displays a succession of high-amplitude peaks rhythmically modulated with a period around 150 ms (about 7 Hz). Although we don’t claim that this is a direct proof of our hypothesis, it shows nonetheless that a rhythmic modulation of HFA in the theta/alpha range exists in sensory areas selective to the kind of information stored in that region (spatial positions in the parietal visual pathway). Such modulation might support the periodic reactivation of memory traces. It remains to be determined whether the modulation of gamma peaks corresponds to a top-down modulation by the PFC or potentially other regions such as the hippocampus (Axmacher et al., 2007), and whether it targets specifically the neural assemblies storing items in memory.

The above model implies that WM capacity can be inferred from attention’s discrete sampling rate together with the decay rate of HFA, both of which are measurable. WM capacity is the number of neural assemblies attention can shift to and reactivate before they fully decay. With a sampling rate of 6–10 Hz, 3–5 items can be sequentially reactivated within 450 ms (the duration of the decay), in line with previous findings on WM capacity (Vogel et al., 2005). This model of WM accounts for the asynchronous firing of neural assemblies, the limited WM capacity, and the involvement of attention and PFC in WM, in addition to the explanatory power of previous proposed mechanisms (Luck and Vogel, 2013). Further, it provides clear and testable predictions: (1) participants who can shift their attention more rapidly should have a higher WM capacity. Indeed, this has been shown by Anderson et al. (2013), who found that individuals with higher WM capacity had more efficient search slopes in a visual search task; and (2) the decay rate of HFA in a particular sensory area (say, the Fusiform Face area) should also predict WM capacity of items maintained in that brain region (say, faces) for that individual.

### Putting it all together

We have suggested a neural implementation of a periodic reactivation-based theory of WM. The prerequisite neural phenomena of such a model are the following:

- precise memory traces of sensory stimuli are stored in cortical regions which can differentiate between nearly identical stimuli (i.e., at the level of different neural representations, to avoid confusion at the time of retrieval)—we argue that this is why traces must be stored in sensory areas.
- once a stimulus is no longer physically present, the activity of its neural representation decays progressively with time.
- once the activity of a neural representation has decayed down to baseline level, we claim that it can no longer be reactivated—a neural representation is active as long as gamma-band activity is above baseline level in its corresponding neural assembly.
- reactivation of decaying memory traces originates from brain regions supporting the instruction to keep those traces active—we propose the dorsal lateral PFC as a likely candidate.
- the reactivation mechanism is able to bring back gamma-band activity of an active neural assembly significantly above baseline level.
- the reactivation mechanism cannot reactivate distinct neural assemblies simultaneously: there is a minimum shift-time between two assemblies.

This neural implementation of WM is conceptually similar to striking a bell with a hammer. In this analogy, the decaying neural representation of a stimulus, in terms of in gamma-band activity, is periodically reactivated by rhythmic attention. If a bell/stimulus decays within 500 ms (see Figure 3) and the hammer/attention takes 100–200 ms to go from one bell/stimulus to the other, 3–5 bells/stimuli can be kept active at the same time. Our account can thus explain a limited WM capacity through the periodicity of attention. It has also closely related to several existing theories of WM.

### Relation to other theories

Another neural model of WM that is similar to our proposed theory is the model developed by Lisman and Idiart (1995), Raffone and Wolters (2001) and Lisman and Jensen (2013). This model assumes that individual items are represented by different cell assemblies that are each active within a different gamma cycle (Fell and Axmacher, 2011). Since gamma cycles have been found to be nested in theta cycles (Lisman and Jensen, 2013) their hypothesis is that the to-be-remembered items are being sequentially reactivated during every theta cycle. The role of gamma is thus to allow alternating assemblies of cells to fire, while the role of theta is to organize these different assemblies sequentially. This theory differs from our proposal in that reactivation of successive assemblies takes about 100–300 ms in our proposal, much slower than the time scale in Lisman et al.’s model, where such reactivations can be estimated to occur every 20–40 ms. The theory by Lisman and Idiart has also been specifically linked to oscillatory coupling between the phase of a lower frequency and the amplitude of a higher frequency (Canolty et al., 2006; Canolty and Knight, 2010). However, our proposal does not make any specific claims about the involvement of theta oscillations or direct coupling between theta and gamma oscillations. It does not need to do so, since the low-frequency modulation refers to a modulation of gamma amplitude itself. Support for this choice comes from earlier studies that have provided some evidence for a lack of phase-amplitude coupling of the sort Lisman-Idiart proposed (Axmacher et al., 2010). Nevertheless, the theta-timescale for reactivation in their model is quite similar to the waxing of waning of attention in our model. The time constant of decay to be used for modeling their data should be empirically testable, by directly looking at decay (and reactivation) rates in successful WM storage of multiple items.

Luck and Vogel (2013), summarizing decades of research, concluded that WM has a “slot-like” nature, where up to four distinct items can be simultaneously stored. They also proposed that, based on Lisman et al.’s work, each item is represented by an active neural ensemble, which is periodically reactivated by local circuits, and hence sustained throughout the maintenance period. However, as we noted, this does not explain how task set can affect which items are stored in WM. Further, Sreenivasan et al. (2014) showed that the PFC is intimately involved in coordinating what items are represented in the sensory areas over time, although it does representing these items itself. This means that local reactivation circuits provide an incomplete picture of WM mechanisms, since they preclude any role for PFC. We combined these two different strands of analyses and suggested that PFC-mediated attention acts on representations in sensory areas and periodically reactivates them, as necessary. Thus, we explain both the role of PFC and the finding that task set can influence WM. Our model is also consistent with the proposal by Barrouillet et al. (2007) which posits that attention plays a crucial role in maintaining items in WM by periodically refreshing them, and thus preventing them from decaying completely. As detailed above, our account also leads to testable predictions about individual differences in WM capacity. Our theory could in the future be tested empirically by measuring the attentional sampling rate for an individual (Chakravarthi and VanRullen, 2011a) at the same time as decay of gamma activity in higher-level sensory areas corresponding to the type of information being maintained. A potentially even stronger test would be a disruption of the proposed refreshing mechanism—which we propose is subserved by interactions between PFC and higher-level sensory areas—for example by stimulation studies.

### Assumptions about speed of visual attention

A possible objection against our proposal is that it depends on a speed of visual attention moving at 6–10 Hz, or 100–200 ms per item. However, the visual search literature potentially provides a very different estimate of the speed of visual attention. Visual search, where observers are asked to report the presence of a particular object among distracter objects, has been used as a measure of attentional processing for several decades (e.g., Treisman and Gelade, 1980; Wolfe, 1998; Eckstein, 2011; Nakayama and Martini, 2011). To successfully search for the target, attention has to process several items sufficiently such that it can discard the distracters and select only the target. These studies have provided estimates of how long attention has to process each item to perform the search task effectively. Such estimates of attentional processing time have ranged from 0–10 ms ms (“pop-out” search) to 100 s of ms (“serial” search). Despite the wide range of search “slopes”, in popular literature these results have been summarized as attention requiring about 25 ms per item. In other words this would suggest that attention processes about 40 items a second, which contradicts the proposal provided here. However, there are details of the visual search paradigm that resolve this apparent contradiction.

A search slope of 25 ms/item does not mean that attention samples each item for 25 ms and rapidly switching between items. It merely means that attention can process an object’s features to a sufficient extent within that period for it to make a decision (target/distracter) about the object. Further, it need not be the case that attention processes each object at the rate of 40 Hz. It could be that attention samples a few features of several objects at much slower periods. Say that attention selects and processes four sets of partial features (belonging to four different objects) every 100 ms. This would give a search slope of 25 ms/item, whereas in fact it has sampled and processed features every 100 ms (i.e., a sampling rate of 10 Hz). Indeed, visual search literature is replete with examples where minor changes among one of many target and distracters attributes dramatically changes search slopes, suggesting that attention does not process entire objects, but only the task relevant features of the objects. For example, distracter and target familiarity (Wang et al., 1994) or target-distracter similarity and homogeneity (D’Zmura, 1991) can considerably affect search slopes. In other words, search slopes are not good measures of how long attention processes a single object. Consequently, the 25 ms/item is probably a misleading number. Indeed, there is an ongoing debate about how “serial” visual search is. If search is not exactly serial, then the slopes cannot be used to estimate attentional processing speeds directly. To overcome this limitation in interpretation, several researchers have devised more sophisticated ways of measuring how long attention stays in one place and samples its contents (e.g., Horowitz et al., 2004, 2009; Carlson et al., 2007) and have consistently found that involuntary attention takes about 100–150 ms to switch locations, consistent with the current claims. In summary, we do not see a contradiction between the visual search literature and our model. Visual search indexes a different set of attentional processes and does not provide a direct estimate of attentional sampling times, as one might be tempted to infer.

## Conclusion

We have suggested a neural implementation of a periodic reactivation theory of WM in sensory areas. We have shown preliminary evidence that parameters such as decay rates for the to-be-remembered items can be read off from gamma-band activity recorded in iEEG. Coupled with the findings that attention samples items at around 6–10 Hz, we provide physiological grounding to the well-established estimate of WM capacity of 3–5 items and some popular theories of WM.

## Conflict of interest statement

The authors declare that the research was conducted in the absence of any commercial or financial relationships that could be construed as a potential conflict of interest.
